# Occult HIV-1 drug resistance to thymidine analogues following failure of first-line tenofovir combined with a cytosine analogue and nevirapine or efavirenz in sub Saharan Africa: a retrospective multi-centre cohort study

**DOI:** 10.1016/S1473-3099(16)30469-8

**Published:** 2016-12-01

**Authors:** John Gregson, Pontiano Kaleebu, Vincent C Marconi, Cloete van Vuuren, MB ChB, Nicaise Ndembi, Raph L Hamers, Phyllis Kanki, Christopher J Hoffmann, Shahin Lockman, Deenan Pillay, Tulio de Oliveira, Nathan Clumeck, Gillian Hunt, Bernhard Kerschberger, Robert W Shafer, Chunfu Yang, Elliot Raizes, Rami Kantor, Ravindra K Gupta

**Affiliations:** Department of Statistics, London School of Hygiene & Tropical Medicine, London, UK (J Gregson PhD); MRC/UVRI Uganda Research Unit on AIDS, Entebbe, Uganda (Prof P Kaleebu PhD); Uganda Research Unit on AIDS, Entebbe, Uganda (Prof P Kaleebu); Department of Global Health, Emory University Rollins School of Public Health (Prof V C Marconi MD) and Division of Infectious Diseases, Emory University School of Medicine (Prof V C Marconi), Emory University, Atlanta, GA, USA; Division of Infectious Diseases, University of the Free State, and 3 Military Hospital, Bloemfontein, South Africa (C van Vuuren MB ChB); Institute of Human Virology Nigeria, Abuja, Nigeria (N Ndembi PhD); Amsterdam Institute for Global Health and Development, Department of Global Health, Academic Medical Center, University of Amsterdam, Netherlands (R L Hamers MD); Department of Immunology and Infectious Disease, Harvard T H Chan School of Public Health, Boston, MA, USA (Prof P Kanki DSc); Aurum Institute, Johannesburg South Africa (C J Hoffmann MD); Johns Hopkins University, Baltimore, MD, USA (C J Hoffmann); Brigham and Women’s Hospital, Boston, MA, USA (S Lockman MD); Department of Infection, University College London, London, UK (Prof D Pillay PhD, Prof R K Gupta FRCP); Africa Health Research Institute, KwaZulu Natal, South Africa (Prof R K Gupta, Prof D Pillay); Nelson R Mandela School of Medicine, School of Laboratory Medicine and Medical Sciences, College of Health Sciences, University of KwaZulu Natal, Durban, South Africa (Prof T de Oliveira PhD); Centre for the AIDS Programme of Research in South Africa (CAPRISA), Durban, South Africa (Prof T De Oliveira); Saint-Pierre University Hospital, Université Libre de Bruxelles, Brussels, Belgium (Prof N Clumeck MD) National Institute for Communicable Diseases, Sandringham, South Africa (G Hunt PhD; Médecins Sans Frontières (Operational Centre Geneva), Mbabane, Swaziland (B Kerschberger MD); Department of Medicine, Stanford University, Stanford, CA, USA (Prof R W Shafer MD); International Laboratory Branch (C Yang PhD), Division of Global HIV/AIDS, Center for Global Health, Centers for Disease Control and Prevention, Atlanta, GA, USA (E Raizes MD); and Division of Infectious Diseases, Alpert Medical School, Brown University, Providence, RI, USA (Prof R Kantor MD)

## Abstract

**Background:**

HIV-1 drug resistance to older thymidine analogue nucleoside reverse transcriptase inhibitor drugs has been identified in sub-Saharan Africa in patients with virological failure of first-line combination antiretroviral therapy (ART) containing the modern nucleoside reverse transcriptase inhibitor tenofovir. We aimed to investigate the prevalence and correlates of thymidine analogue mutations (TAM) in patients with virological failure of first-line tenofovir-containing ART.

**Methods:**

We retrospectively analysed patients from 20 studies within the TenoRes collaboration who had locally defined viral failure on first-line therapy with tenofovir plus a cytosine analogue (lamivudine or emtricitabine) plus a non-nucleoside reverse transcriptase inhibitor (NNRTI; nevirapine or efavirenz) in sub-Saharan Africa. Baseline visits in these studies occurred between 2005 and 2013. To assess between-study and within-study associations, we used meta-regression and meta-analyses to compare patients with and without TAMs for the presence of resistance to tenofovir, cytosine analogue, or NNRTIs.

**Findings:**

Of 712 individuals with failure of first-line tenofovir-containing regimens, 115 (16%) had at least one TAM. In crude comparisons, patients with TAMs had lower CD4 counts at treatment initiation than did patients without TAMs (60·5 cells per μL [IQR 21·0–128·0] in patients with TAMS *vs* 95·0 cells per μL [37·0–177·0] in patients without TAMs; p=0·007) and were more likely to have tenofovir resistance (93 [81%] of 115 patients with TAMs *vs* 352 [59%] of 597 patients without TAMs; p<0·0001), NNRTI resistance (107 [93%] *vs* 462 [77%]; p<0·0001), and cytosine analogue resistance (100 [87%] *vs* 378 [63%]; p=0·0002). We detected associations between TAMs and drug resistance mutations both between and within studies; the correlation between the study-level proportion of patients with tenofovir resistance and TAMs was 0·64 (p<0·0001), and the odds ratio for tenofovir resistance comparing patients with and without TAMs was 1·29 (1·13–1·47; p<0·0001)

**Interpretation:**

TAMs are common in patients who have failure of first-line tenofovir-containing regimens in sub-Saharan Africa, and are associated with multidrug resistant HIV-1. Effective viral load monitoring and point-of-care resistance tests could help to mitigate the emergence and spread of such strains.

## Introduction

Combination antiretroviral therapy (cART) can lead to declining mortality and HIV incidence in high prevalence settings.^1,2^ Virological failure occurs after 12 months in 15–35% of patients treated with thymidine analogue-containing first-line regimens (eg, zidovudine or stavudine plus lamivudine plus nevirapine or efavirenz), with most cases of resistance to non-nucleoside reverse transcriptase inhibitors (NNRTIs) and lamivudine occurring in regions without access to routine viral load monitoring.^3,4^ HIV-1 drug resistance could be responsible for nearly 425 000 AIDS-related deaths and 300 000 new infections over the next 5 years.^5^

WHO has recommended first-line tenofovir disoproxil fumarate (tenofovir) instead of thymidine analogues since 2012.^6^ Of the 17 million people accessing first-line ART in 2016,^7^ roughly 3·5 million were treated with a thymidine analogue.^8^ During the process of programmatic tenofovir substitution in ART-treated individuals (including children), confirmation of viral suppression before the regimen change (within 30 days) is rarely done in sub-Saharan Africa because of poor access to viral load testing. Given the potential substantial prevalence of unrecognised virological failure and drug resistance in this setting,^4,9–11^ programmatic single-drug substitutions risk more rapid acquisition of high-level drug resistance not only to NNRTIs and cytosine analogues, but also to tenofovir.^12^ Importantly, NNRTI resistance and thymidine analogue resistance mutations (TAMs) can be transmitted to uninfected individuals who are subsequently at increased risk of ART failure themselves.^13^

**Research in context**

**Evidence before this study**

We did a systematic review using PubMed and Embase, searching from Jan 1, 2000, up to Aug 15, 2016, without language limitations. Manuscripts of interest were also identified from the reference lists of selected papers, clinical trials registries, and abstracts from the Conference on Retroviruses and Opportunistic Infections (CROI) and International AIDS Society (IAS). We used the search terms “HIV” AND “Tenofovir” AND “thymidine analogue” OR “stavudine” OR “zidovudine” OR “AZT” OR “d4T”. We found no studies reporting the implications of previous thymidine analogue use on outcomes following tenofovir-based antiretroviral therapy (ART). One study investigated the implications of transition from thymidine analogue to tenofovir by use of a cross sectional survey in Myanmar before the introduction of tenofovir. The investigators tested viral loads in more than 4000 patients after 12 months of thymidine analogue-based ART to avoid substitutions in viraemic patients. They noted that a substantial proportion of patients were having treatment failure (13% had viral loads >250 copies per mL), in whom direct tenofovir substitution for the thymidine analogue would not be appropriate.

**Added value of this study**

Our results show that tenofovir-based first-line regimens are failing in a substantial proportion of patients who have evidence of previous exposure and drug resistance to older nucleoside (thymidine) analogues such as zidovudine and stavudine in sub-Saharan Africa. These individuals are likely to have developed drug resistance to the non-nucleoside reverse transcriptase inhibitor as well as the cytosine analogue, and therefore have high-level resistance to at least two of the three drugs present in tenofovir-based first line ART. Our data show that these individuals with thymidine analogue mutations have lower CD4 counts and therefore are at greater risk of clinical complications than are those without previous ART exposure.

**Implications of all the available evidence**

Cheap and effective viral load monitoring, resistance testing, or both could prevent the transition of patients with virological failure onto tenofovir-based first-line ART and also identify individuals with pre-existing drug resistance to first line agents arising from undisclosed prior ART. These individuals could then be treated with second-line regimens.

A further complication to the introduction of tenofovir in sub-Saharan Africa is shown by data suggesting that individuals presenting as treatment naive often do not disclose previous ART exposure, which is most likely with thymidine analogue-based ART.^14^ Accordingly, we have previously reported unexplained TAMs in patients after viral failure of tenofovir-containing first-line regimens.^15^ In this Article, we characterise the prevalence, determinants, and implications of TAMs in patients after virological failure of tenofovir-containing first-line regimens in sub-Saharan Africa.

## Methods

### Study population and design

We identified patients from within the TenoRes collaboration, a multicountry retrospective study examining correlates of genotypic drug resistance following failure of tenofovir-containing combination ART. Data in this report cover seven countries with baseline measurements taken between 2005 and 2013. The original TenoRes collaboration spans 36 counties with baseline measurements between 1998 and 2015. Our methods have been described previously.^15^ Briefl y, we collected data from cohorts with documented virological failure after first-line ART consisting only of tenofovir plus either lamivudine or emtricitabine plus either efavirenz or nevirapine, with no previously known exposure to additional nucleoside reverse transcriptase inhibitors such as zidovudine or stavudine (appendix). Virological failure was defined as a viral load greater than 1000 copies per mL, except for two studies in which the definition was viral load greater than 2000 copies per mL (appendix). Patients needed to have had a successful resistance test result associated with virological failure of combination ART and been on tenofovir-based ART for a minimum of 4 months before virological failure. We collected information on baseline characteristics (age, sex, pre-tenofovir CD4 count, pre-tenofovir viral load, and previous exposure to single-dose nevirapine for prevention of vertical transmission), and HIV genotype following virological failure (eg, number and type of TAMs; presence of cytosine analogue, tenofovir, or NNRTI [ie, nevirapine and efavirenz] resistance). In our previous report,^15^ we excluded patients with TAMs because of concerns that they might represent pre-treated rather than first-line patients, although identical information was collected on patients irrespective of the presence or absence of TAMs at the resistance test.

We defined tenofovir resistance as the presence of Lys65Arg/Asn or Lys70Glu/Gly/Gln mutations in reverse transcriptase. Although the presence of three or more TAMs inclusive of either the Met41Leu or Leu210Trp mutation has also been shown to compromise tenofovir clinically,^12^ no individuals in this study had such a profile. TAMs were defined as Met41Leu, Asp67Asn, Lys70Arg, Leu210Trp, Thr215Phe/Tyr, or Lys219Gln/Glu. Our definition of TAMs also included the revertant mutations Thr215Ser/Cys/Asp/Glu/Ile/Val, although only two patients presented with such a mutation without the presence of at least one other TAM. TAM revertants are indicative of previous TAM Thr215Phe or Thr215Tyr mutations in the individual, and have been associated with increased risk of treatment failure if a thymidine analogue drug is used.^16^ We restricted our analysis to study sites from sub-Saharan Africa because we specifically wanted to investigate the large-scale programmatic shifts in tenofovir use that are currently occurring in this region in the absence of intensive viral load monitoring and baseline resistance testing. Studies were included if they had resistance data on ten or more patients, although in sensitivity analyses that included all available data, the conclusions were not altered (appendix).

We interpreted drug resistance mutations using the Stanford HIV Drug Resistance Algorithm version 7.0.

### Statistical analysis

In cohorts spanning multiple countries, each country within the cohort was treated as a separate study for the purposes of our meta-analyses, to ensure that within-study associations were not confounded by between-country differences. To compare baseline characteristics according to TAM resistance, we used Mann-Whitney *U* tests or χ^2^ tests. We did three main analyses. First, we calculated prevalence estimates within each study separately and used Clopper-Pearson exact 95% CIs. Second, we graphically compared the study-level prevalence of TAMs and other drug-resistance mutations and used Spearman’s rank correlation coefficients to assess the strength of association between the two. Third, we calculated odds ratios for drug-resistance mutations in patients with and without TAMs. We pooled estimates across studies using fixed-effects meta-analyses with Mantel-Haenszel weighting. We chose this strategy because there was no evidence of any between-study heterogeneity, and Mantel-Haenszel weighting works well in scenarios with zero-cell counts. All analyses were done with STATA version 11.2.

### Role of the funding source

The funders of the study had no role in study design, data collection, data analysis, data interpretation, or writing of the report. RKG and JG had full access to all the data in the study and had final responsibility for the decision to submit for publication.

## Results

We assessed 34 studies and excluded 14 because they contained fewer than ten patients (56 patients excluded). We identified 712 patients who had viral failure with WHO-recommended, tenofovir-based first-line regimens in 20 studies across sub-Saharan Africa (table 1; appendix). Most (461 [65%]) patients were from southern Africa, with 159 (22%) patients from eastern Africa and 92 (13%) from west and central Africa. 481 (68%) of 712 infections were with HIV-1 subtype C (appendix). Median age at baseline was 35·0 years (IQR 28·8–40·7) and 413 (58%) patients were women. The median year of initiation was 2011, and patients were followed up for a median of 18 months (12–27). Where available, the overall median baseline CD4 count was 92 cells per μL (34–169) and median viral load was log_10_ 5·23 copies HIV-1 RNA (4·5–5·6) per mL. Patient characteristics were broadly similar between patients with and without TAMs, with the exception of baseline CD4 count, which was roughly 30 cells per μL lower in patients with TAMs in all regions (p=0·007). We noted that usage of emtricitabine was 10% lower in patients with TAM compared to those without. 33 (16%) of 209 women with available data on single-dose nevirapine had known previous exposure to single-dose nevirapine. Prevalence of NNRTI resistance was 88% (29 of 33 patients) in patients with single-dose nevirapine exposure and 82% (378 of 462 patients overall or 142 [81%] of 176 women) in those without single-dose nevirapine exposure (p=0·38). For many patients, it was not known whether or not they had received single-dose nevirapine, including men, for whom single dose nevirapine use was always answered as no.

**Figure 1:**
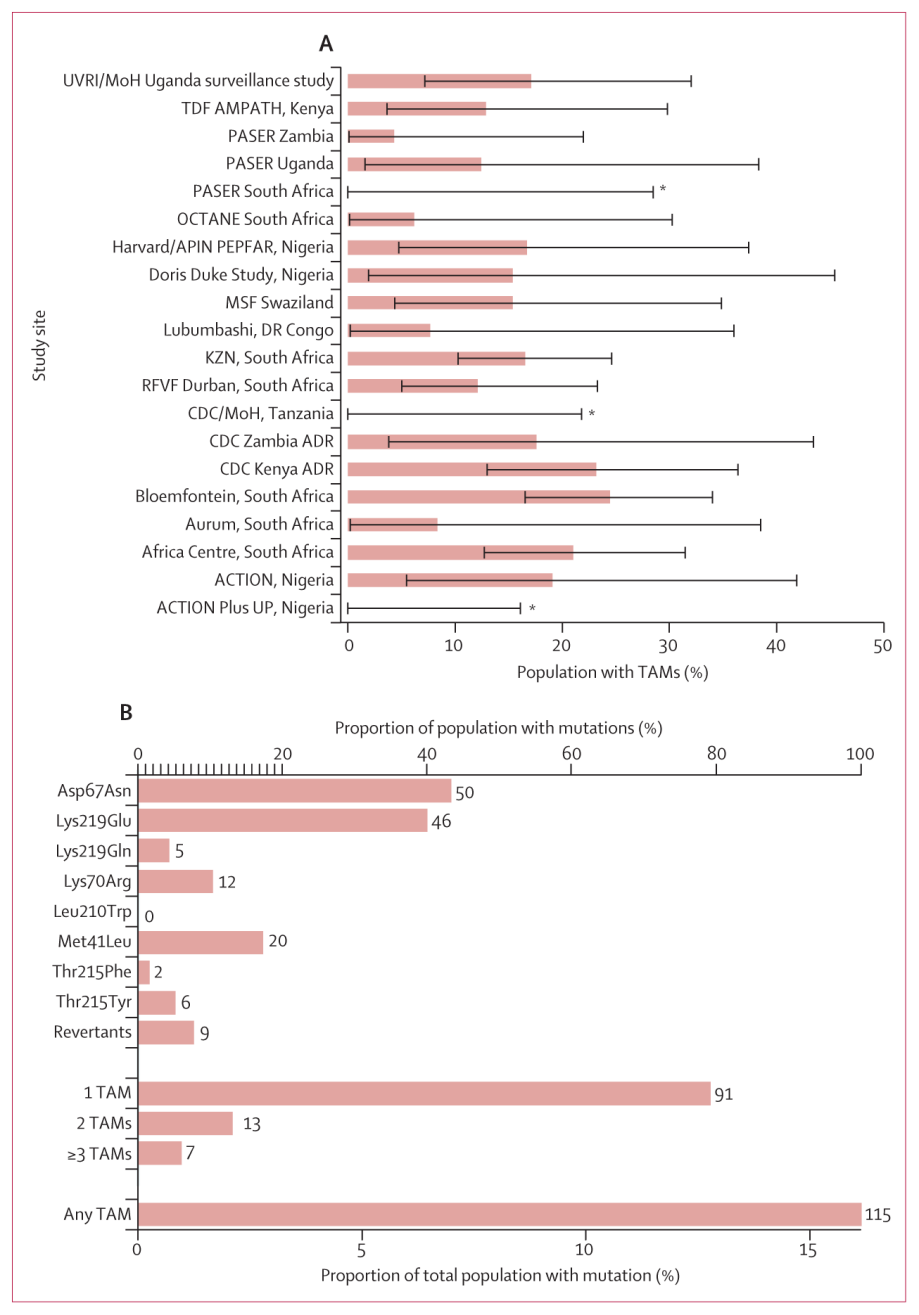
Estimated prevalence of TAMs and types of TAMs (A) Estimated prevalence of TAMs by study site. Black lines show 95% CIs for estimated prevalence. (B) Number and type of TAMs identified across study sites. TAM=thymidine analogue mutation. *Prevalence estimate of 0% where the 95% CI uses the population size and the fact that no TAMs have occurred to put an upper limit on the estimated prevalence.

TAMs were detected in 115 (16%) of 712 patients (figure 1A). The prevalence of TAMs was similar in eastern Africa (26 [16%] of 158), southern Africa (78 [17%] of 461 patients), and west and central Africa (11 [12%] of 92 patients). TAMs were less common in patients with HIV-1 subtype D than in patients with other subtypes (appendix). Despite individual studies tending to have only a small number of patients, all but four of the 20 included studies reported a prevalence of TAMs between 5% and 25% (figure 1A). Asp67Asn was the most common TAM and was present in 50 (7%) of 712 patients; it was more common in southern (41 [9%] of 461 patients) and eastern Africa (eight [5%] of 159 patients) than in west and central Africa (one [1%] of 91 patients; p=0·015). The next most common TAMs were Lys219Glu (46 [6%] of 712 patients) and Met41Leu (20 [3%] patients; figure 1B). 20 (3%) patients had two or more TAMs and seven (1%) patients had three or more TAMs.

**Figure 2:**
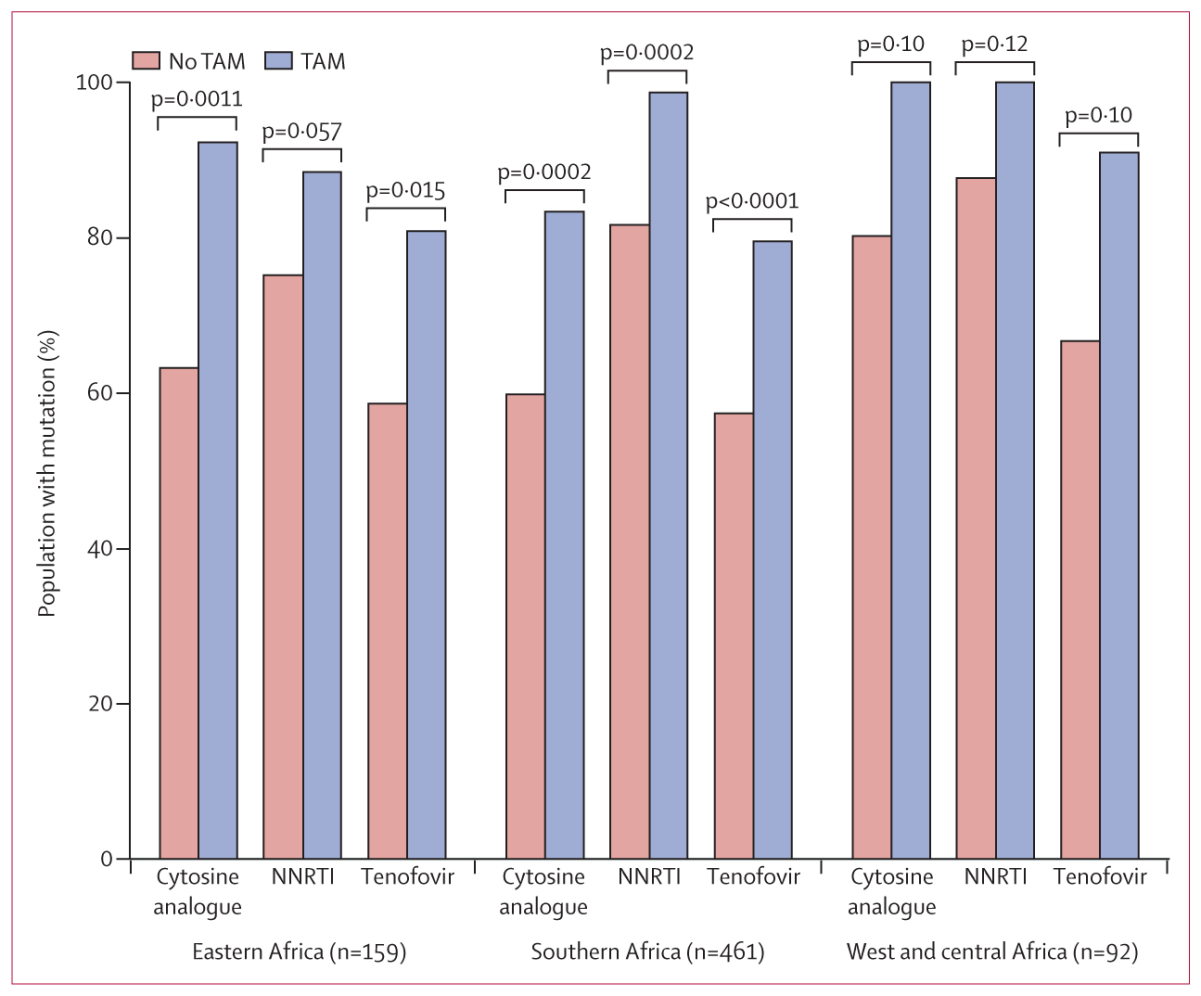
Estimated prevalence of drug resistance mutations Prevalence of resistance to nevirapine or efavirenz (NNRTIs), tenofovir, and cytosine analogue by presence or absence of TAM mutations. TAM=thymidine analogue mutation. Tenfovir=tenofovir disoproxil fumarate. NNRTI=non-nucleoside reverse transcriptase inhibitor.

In crude comparisons across the entire study population, patients with TAMs were more likely to have tenofovir resistance (p<0·0001), as well as resistance to cytosine analogues (100 [87%] patients with TAMs *vs* 378 [63%] of patients without TAMs; p=0·0002) and nevirapine or efavirenz (107 [93%] of 115 patients with TAMs *vs* 462 [77%] of 597 without TAMs; p<0·0001), with consistent findings across all regions (figure 2). Of the 115 patients with TAMs, 93 (81%) had Lys65Arg/Asn or Lys70Glu/Gly/Gln, whereas in the remaining 597 patients without TAMs, 352 (59%) patients had these tenofovir resistance mutations (p<0·001). Tenofovir resistance mutations at Lys65 or Lys70 were present in 92 (86%) of 107 patients with TAM mutations without Thr215Phe/Tyr, and one (13%) of eight patients with TAM mutations with Thr215Phe/Tyr (p<0·0001).

**Figure 3:**
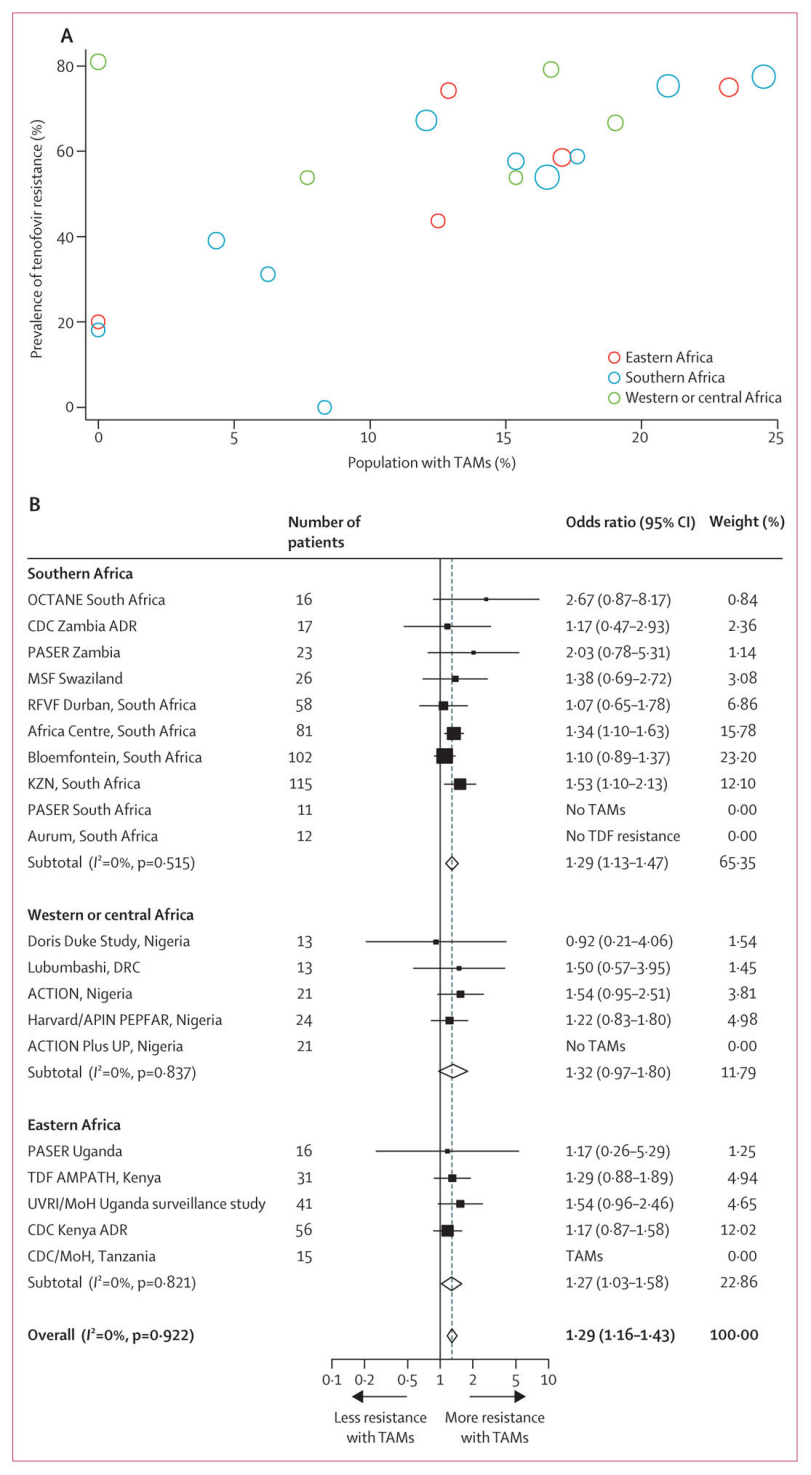
Study-level prevalence of TAMs and association with tenofovir resistance (A) Scatter plot of study-level prevalence of tenofovir resistance and prevalence of TAMs by region. Markers are weighted by study size. (B) Meta-analysis of odds ratios for tenofovir resistance in participants with TAMs versus those without TAMs within individual studies. TAM=thymidine analogue mutation. Tenfovir=tenofovir disoproxil fumarate.

We found a significant association between TAMs and tenofovir resistance both at the study-level and the individual-level. Studies with the highest prevalence of TAMs tended to also have the most tenofovir resistance (figure 3A, Spearman’s ρ of study-level resistance was 0·64, p<0·0001). For example, in the ten studies in which less than 15% of patients had TAMs, tenofovir resistance was present in 112 (52%) of 216 patients, whereas in the ten studies with more than 15% of patients with TAMs, tenofovir resistance was present in 333 (67%) of 496 patients (p<0·0001). We found similar associations for other drug resistance mutations, such as higher levels of nevirapine or efavirenz resistance and cytosine analogue resistance in patients with TAMs (appendix).

**Figure 4:**
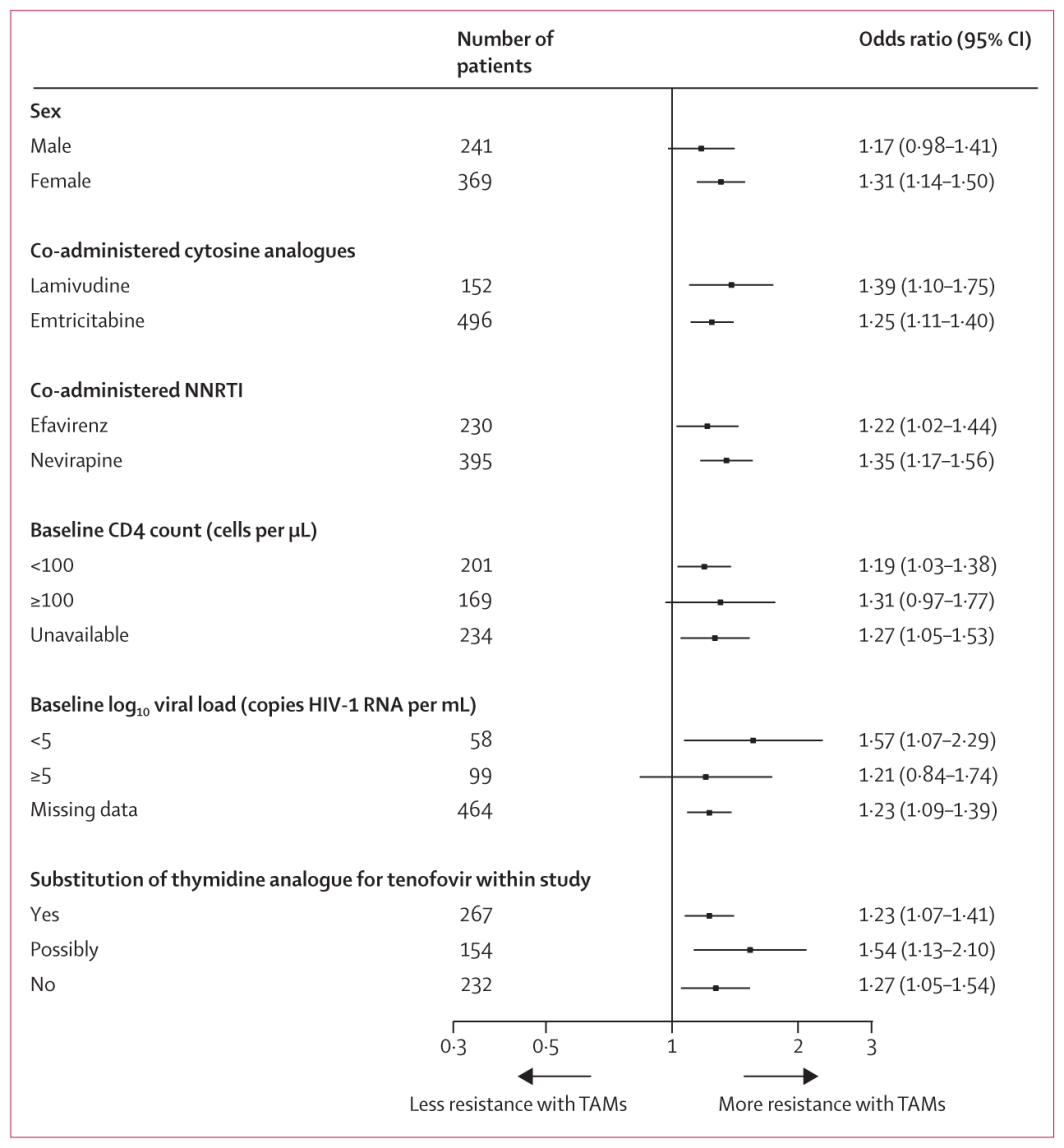
Effect of TAMs on tenofovir resistance in subgroups of patients Within-study odds ratios for tenofovir resistance by presence or absence of TAMs, stratified by baseline characteristics. TAM=thymidine analogue mutation. Tenfovir=tenofovir disoproxil fumarate. NRTI=nucleoside reverse transcriptase inhibitor. NNRTI=non-nucleoside reverse transcriptase inhibitor.

Within the study, patients with a TAM were more likely to also have tenofovir resistance (odds ratio 1·29, 95% CI 1·16–1·43; figure 3B). The association was maintained among patients stratified by co-administered cytosine analogue, co-administered nevirapine or efavirenz, sex, baseline viral load (<log_10_5 copies per mL *vs* ≥log_10_5 copies per mL), or baseline CD4 count (<100 cells per μL *vs* ≥100 cells per μL; figure 4). Notably, OR for tenofovir resistance was not affected by the possibility of within study drug substitution of thymidine analogue for tenofovir (figure 4). We found similar, although slightly weaker, within-study associations of TAM mutations with both nevirapine or efavirenz resistance and cytosine analogue resistance (appendix).

We assessed studies for potential within-programme drug substitutions and whether viral load confirmation was sought beforehand (table 2). We found that thymidine analogue substitution for tenofovir had occurred and that suppression was rarely confirmed before the change in treatment. Three studies implemented resistance testing before initiating tenofovir, although none excluded patients with drug resistance from initiating first line ART.

## Discussion

We found TAMs that are specifically selected by zidovudine or stavudine in roughly 16% of patients with failure of tenofovir-based first-line antiretroviral regimens. TAMs were associated with greater drug resistance to all components of WHO recommended, tenofovir-containing first-line treatment. The prevalence of resistance to tenofovir reached 80% in individuals with TAMs, a result that is concerning and very much unexpected given that the tenofovir mutation Lys65Arg and TAMs are thought to be antagonistic to one another.^17^ Patients with TAMs tended to have lower CD4 counts than did patients without TAMs, which is consistent with longer duration of infection or faster disease progression.

Our drug resistance prevalence estimates represent prevalence for participants with documented virological failure. Although it is important to know the prevalence of drug resistance among all participants treated with first-line therapy, this was not possible, mostly because of the absence of a clear denominator in many sites. A large international meta-analysis^11^ reported that 15–35% of patients initiating ART in sub-Saharan Africa have virological failure by 12 months. In view of our prevalence estimate of 16% of patients with virological failure having TAMs, we estimate that between 2% and 6% of individuals treated with tenofovir plus cytosine analogue plus efavirenz will have TAMs and 2–5% would have drug resistance to thymidine analogues, tenofovir, cytosine analogues, and the NNRTIs nevirapine and efavirenz within 1 year of treat ment initiation under current practices in sub-Saharan Africa. As previously reported,^15^ an additional 8–18% of patients are likely to have resistance to tenofovir, cytosine analogues, and NNRTIs, but without thymidine analogue resistance.

There are three possible sources of TAMs in patients on first-line tenofovir. The first is transmitted drug resistance, which is unlikely to account for the majority of cases in this study because transmitted drug resistance of TAMs is rare (<1% of TAMs in untreated patients result from being transmitted).^18,19^ Additionally, TAMs and Lys65Arg are antagonistic at the level of the viral genome;^17^ our findings showing co-existence of TAMs and Lys65Arg in patients with virological failure possibly result from these mutations occurring on different viral genomes after sequential therapies. Because transmission is usually with a single viral variant, transmitted drug resistance with TAM would translate to a viral population within an individual that consists entirely of TAM-containing viruses (or reversion variants). Under this scenario antagonism with Lys65Arg would be active and we would therefore not expect to see Lys65Arg and TAMs together in the same individuals.

The second possibility is programmatic substitution, wherein tenofovir was used to replace a thymidine analogue at a time when the patient had occult treatment failure. Under this scenario the most likely sequence of events would be, first, acquisition of cytosine analogue resistance, TAM, and NNRTI mutations during prolonged viral failure, followed by a switch to tenofovir and subsequent emergence of Lys65Arg that confers tenofovir resistance. Therefore, prevention of the develop ment of Lys65Arg mutation could only be achieved by viral load suppression confirmation before the switch in treatments. Effective viral load monitoring has been identified as a priority area^20^ and would trigger adherence counselling and then a possible switch to a second-line protease inhibitor-based regimen instead of continuation of a failing first-line regimen with the substitution of a thymidine analogue for tenofovir. A large study in Myanmar (where tenofovir substitution is planned) has monitored viral loads in more than 4000 patients after 12 months of thymidine analogue-based ART, with the aim of avoiding substitutions in viraemic patients. The investigators found that 13% of patients had viral loads greater than 250 copies per mL, which was halved after adherence counselling was done, reinforcing the need for viral load monitoring before drug substitution.^21^

However, the second scenario cannot account for many of the TAMs identified in the present study because we detected TAMs in cohorts in which no programmatic substitution had occurred and tenofovir-based ART was used at the outset in apparently untreated patients (table 2).^22,23^ The third possibility, which we believe could account for most of the TAMs in the present study is previous undisclosed ART use with undocumented viral failure and drug resistance. This hypothesis is supported by the lower CD4 counts detected in patients with TAMs. Moreover, significant variation has been reported in viral load monitoring practices between rural and urban settings in South Africa,^24^ possibly explaining how unrecognised viral failure and drug resistance during tenofovir substitution could occur in settings where viral load monitoring is centrally funded and part of national guidelines.

To prevent drug resistance due to undisclosed previous ART use, accessible point-of-care baseline resistance screening^25^ could be used to assist in the identification of patients with resistance to the components of first-line ART. We have previously identified key mutations that could be used in such assays, including Lys65Arg, Lys103Asn, Val106Met, Tyr181Cys, Gly190Ala, and Met184Val,^26^ and on the basis of the present study, Asp67Asn and Lys219Gln/Glu could be added to this list. If HIV-1 drug resistance is detected with such assays, second-line ART could be initiated, taking into account the mutations identified. If they become sufficiently cheap and reliable, drug resistance assays could be used in place of viral load monitoring at treatment initiation or switches.

Our study has some limitations. The sampling was not systematic and therefore prevalence estimates might not be fully representative of countries and regions. Our drug resistance prevalence estimates represent prevalence for participants with documented virological failure. We can only estimate the overall number initiating treatment, because it was not systematically assessed. Using data from WHO and Uganda on the prevalence of virological failure,^11,23^ we calculate that if 15% of people initiating ART have failure at 1 year (on treatment analysis), then our data represent about 4750 patients initiating tenofovir-based first-line ART.

Although none of the studies overtly used targeted viral load testing in individuals suspected of having treatment failure, such targeting might have occurred at the clinical level, potentially biasing our estimates of TAM resistance upwards. Conversely, Sanger sequencing can miss drug resistance mutations in 30% or more of patients.^27^ Additionally, we did not assess thymidine analogue resistance conferred by mutations in the connection domain between HIV-1 reverse transcriptase and RNAseH that are known to be selected by zidovudine,^28^ leading to further underestimation of drug resistance.

Notably, stavudine selects not only for TAMs, but also for Lys65Arg in up to 20% of patients who have failure of stavudine.^9,29–31^ However, given that TAM and Lys65Arg are not selected together by a single stavudine-based regimen,^9,29,32^ exposure to stavudine would probably not explain the genotypes with both TAM and Lys65Arg that were seen in our study.

This study has important policy implications for the limitation of drug resistance as tenofovir becomes more widely used both as treatment^8^ and pre-exposure prophylaxis.^33^ First, a single point-of-care viral load test could be implemented to prevent substitution of first line zidovudine for tenofovir in patients with virological failure. Regular viral load monitoring has been advocated in the past for treatment monitoring and could identify early virological failure in patients with previously undisclosed ART and drug resistance. However, this regular monitoring might be less cost effective than targeted viral load measurement. Second, simple resistance test kits could both assist in screening for drug resistance before ART initiation and also contribute to population level surveillance of HIV-1 drug resistance^25^ in both treated and untreated populations—a priority in sub Saharan Africa given the substantial mortality now recognised to be associated with HIV-1 drug resistance.^5^ These proposals should be part of a multipronged approach and subjected to cost effectiveness assessment in the wider context of other interventions that aim to limit burden of the HIV epidemic.

## Supplementary Material

Supplementary data

## Figures and Tables

**Table 1:** Baseline characteristics of patients by region and thymidine analogue mutation status

	Number of patients	Age at tenofovir initiation (years)	Women	Nevirapine	Emtricitabine	Baseline CD4 count (cells per μL)	Baseline viral load (log_10_ copies per mL)	Year of tenofovir initiation	Length of time on tenofovir-based ART (months)
**Eastern Africa**									

No TAM	133	36·0 (30·0–44·0)	77 (58%)	79 (59%)	44 (33%)	102·5 (40·5–208·5)	5·6 (5·3–5·8)	2011 (2010–2012)	14·2 (12·2–27·8)
TAM	26	33·5 (27·0–41·0)	17 (65%)	20 (77%)	7 (27%)	68·0 (16·5–209·0)	5·4 (5·1–5·8)	2011 (2011–2012)	13·3 (11·8–27·2)

**Southern Africa**									

No TAM	383	34·5 (28·0–41·0)	225 (59%)	96 (25%)	72 (19%)	98·0 (39·0–167·0)	4·7 (3·4–5·4)	2011 (2008–2011)	19·0 (12·0–28·2)
TAM	78	34·0 (28·4–37·0)	48 (62%)	13 (17%)	7 (9%)	72·0 (20·0–107·0)	4·4 (2·9–5·3)	2010 (2010–2011)	21·0 (14·1–27·3)

**West and central Africa**									

No TAM	81	36·1 (31·0–40·0)	42 (52%)	53 (65%)	65 (80%)	86·5 (30·0–180·0)	5·2 (4·9–5·6)	2006 (2006–2009)	14·2 (10·9–18·0)
TAM	11	36·3 (30·0–42·0)	4 (36%)	9 (82%)	9 (82%)	58·0 (27·0–143·0)	4·8 (3·7–5·5)	2006 (2006–2006)	12·4 (11·6–18·0)

**Overall**									

No TAM	597	35·0 (29·0–41·0)	344 (58%)	228 (38%)	181 (30%)	95·0 (37·0–177·0)	5·2 (4·5–5·6)	2011 (2008–2011)	17·4 (12·0–27·0)
TAM	115	34·0 (28·0–38·1)	69 (60%)	42 (37%)	23 (20%)	60·5 (21·0–128·0)	5·1 (4·1–5·6)	2011 (2010–2012)	19·0 (12·9–27·0)

Data are median (IQR) or n (%) unless specified otherwise. Tenfovir=tenofovir disoproxil fumarate. ART=antiretroviral therapy.

**Table 2:** Study-level information on implementation of tenofovir-based ART

	Substitution of thymidine analogue for tenofovir within study?	Virus suppression always confirmed before substitution?	Baseline resistance testing?	Exclusion of patients with baseline resistance?	Possibility of previous undisclosed ART
**Eastern Africa**					

PASER Uganda	Possibly	No	No	NA	Yes
CDC Kenya ADR	No	No	No	NA	Yes
TDF AMPATH, Kenya	No	No	No	NA	Yes
UVRI/MoH Uganda surveillance study	No	NA	Yes	No	Yes
CDC/MoH, Tanzania	Possibly	No	No	No	Yes

**West and central Africa**					

ACTION, Nigeria	Yes	No	No	NA	Yes
ACTION Plus UP, Nigeria	Possibly	No	No	No	Yes
Harvard/APIN PEPFAR	Yes	No	No	NA	Yes
Doris Duke Study, Nigeria	Yes	No	No	NA	Yes
Lubumbashi, DR Congo	No	NA	Yes	No	Yes

**Southern Africa**					

PASER Zambia	Possibly	Not always	No	NA	Yes
PASER South Africa	Possibly	Not always	No	NA	Yes
Africa Centre, South Africa	Yes	Not always	No	NA	Yes
Aurum, South Africa	Yes	Not always	No	NA	Yes
Bloemfontein, South Africa	Yes	Not always	No	NA	Yes
KZN, South Africa	Yes	Not always	No	NA	Yes
MSF Swaziland	Yes	No	No	NA	Yes
OCTANE South Africa	No	NA	Yes	No	Yes
CDC Zambia ADR	No	No	No	NA	Yes
RFVF Durban, South Africa	Possibly	No	No	No	Yes

ART=antiretroviral therapy. NA=not applicable. Tenfovir=tenofovir disoproxil fumarate.
