# Supplementary material for: Occult HIV-1 drug resistance to thymidine analogues following failure of first-line tenofovir combined with a cytosine analogue and nevirapine or efavirenz in sub Saharan Africa: a retrospective multi-centre cohort study
Source: Lancet Infect Dis. Author manuscript; Available in PMC 2017 May 8. (PMC5421555; doi:10.1016/S1473-3099(16)30469-8)
Supplement: Supplementary data [file NIHMS855948-supplement-Supplementary_data.pdf]

THE LANCET Infectious Diseases

Supplementary webappendix

This webappendix formed part of the original submission and has been peer reviewed. We post it as supplied by the authors.

Supplement to: Gregson J, Kaleebu P, Marconi VC, et al. Occult HIV-1 drug resistance to thymidine analogues following failure of first-line tenofovir combined with a cytosine analogue and nevirapine or efavirenz in sub Saharan Africa: a retrospective multi-centre cohort study. *Lancet Infect Dis* 2016; published online Nov 30. http://dx.doi.org/10.1016/S1473-3099(16)30469-8.

Supplementary Table 1: Characteristics of studies from the TenoRes collaboration included in the present analysis

Study	Country	Income region	Study type	Underlying cohort exclusively first line treated?	Follow-up Active* or passive	N	TDF resistance	VL threshold for genotype	Use of FTC	Use of NVP	Baseline CD4 <100	Baseline viral load >100,000
**Sub Saharan Africa**												
**ACTION**	Nigeria	LMIC	Cohort	Yes	Passive	17	10	1000	17 (100%)	7 (41%)	10 (59%)	-
**ACTION Plus UP,**	Nigeria	LMIC	Cohort	Yes	Passive	21	17	1000	18 (86%)	12 (57%)	8 (38%)	-
**Doris Duke Study**	Nigeria	LMIC	Trial	Yes	Active	13	8	1000	0 (0%)	3 (23%)	5 (38%)	7 (54%)
**Harvard/APIN PEPFAR**	Nigeria	LMIC	Cohort	No	Active	20	15	2000	18 (90%)	19 (95%)	16 (80%)	17 (85%)
**CDC Nigeria ADR**	Nigeria	LMIC	Cohort	Yes	Passive	6	3	1000	5 (83%)	6 (100%)	2 (33%)	4 (67%)
**Lubumbashi,**	DRC	LIC	Trial	Yes	Active	12	6	1000	12 (100%)	12 (100%)	7 (58%)	9 (75%)
**UVRI/MoH Uganda surveillance study**	Uganda	LIC	Cohort	Yes	Passive	35	19	1000	18 (51%)	24 (69%)	18 (51%)	29 (83%)
**CDC Uganda ADR**	Uganda	LIC	Cohort	Yes	Passive	5	3	1000	4 (80%)	3 (60%)	2 (40%)	-
**CDC/MoH, Tanzania**	Tanzania	LIC	Cohort	No	Active	15	3	1000	12 (80%)	1 (7%)	-	-
**CDC Kenya ADR**	Kenya	LMIC	Cohort	Yes	Passive	43	31	1000	1 (2%)	27 (63%)	17 (40%)	-
**TDF AMPATH**	Kenya	LMIC	Cohort	Yes	Active	27	19	1000	0 (0%)	23 (85%)	-	-
**PASER**	Nigeria, Uganda, South Africa, Kenya, Zambia, Zimbabwe	LMIC	Cohort	No	Active	53	19	1000	52 (98%)	17 (32%)	27 (51%)	35 (66%)
**Aurum, KZN**	South Africa	HMIC	Cohort	No	Active	11	0	1000	9 (82%)	3 (27%)	1 (9%)	0 (0%)
**Africa Centre, KZN**	South Africa	HMIC	Cohort	No	Passive	64	45	1000	0 (0%)	10 (16%)	32 (50%)	-
**Bloemfontein,**	South Africa	HMIC	Cohort	No	Passive	78	59	1000	2 (3%)	16 (21%)	14 (18%)	1 (1%)
**RFVF, Durban**	South Africa	HMIC	Cohort	Yes	Passive	51	34	1000	0 (0%)	7 (14%)	26 (51%)	0 (0%)
**CDC/NCID, KZN,**	South Africa	HMIC	Cohort	Yes		98	49	1000	0 (0%)	33 (34%)	-	-
**MSF**	Swaziland	HMIC	Cohort	No	Active	22	12	1000	0 (0%)	5 (23%)	10 (45%)	6 (27%)
**CDC Zambia ADR**	Zambia	LMIC	Cohort	No	Passive	14	8	1000	13 (93%)	1 (7%)	4 (29%)	-
**OCTANE**	Kenya, Botswana, Malawi, South Africa, Zambia, Zimbabwe	LMIC	Trial	Yes	Active	36	7	2000	36 (100%)	36 (100%)	16 (44%)	27 (75%)

Supplementary Table 2: Number of patients with available data for covariates and number of patients contributing to subgroup analyses.

Study	N	N with information on CD4 or viral load		Patients contributing to subgroup analyses											
				NRTI		Gender		NNRTI		Baseline CD4 (cells/mm3)			Viral load (log10 HIV1-RNA/ml)		
		Base-line CD4	Base-line viral load	3TC	FTC	Female	Male	EFV	NVP	Unavail-able	<100	>100	Unavai-lable	<5	>5
**Eastern Africa**	**159**	**108**	**57**	**38**	**104**	**76**	**62**	**98**	**42**	**36**	**55**	**53**	**87**	**8**	**48**
CDC Kenya ADR	56	51	0	0	55	36	20	36	20	5	24	27	56	0	0
CDC/MoH, Tanzania	15	0	0	0	0	0	0	0	0	0	0	0	0	0	0
PASER Uganda	16	16	16	15	0	0	10	6	10	0	9	7	0	0	15
TDF AMPATH, Kenya	31	0	0	0	31	18	13	27	0	31	0	0	31	0	0
UVRI/MoH Uganda surveillance study	41	41	41	23	18	22	19	29	12	0	22	19	0	8	33
**Southern Africa**	**461**	**248**	**101**	**58**	**379**	**266**	**146**	**82**	**333**	**198**	**109**	**96**	**356**	**38**	**22**
Africa Centre, South Africa	81	71	0	0	81	62	19	0	70	0	42	29	81	0	0
Aurum, South Africa	12	11	11	0	0	0	0	0	0	0	0	0	0	0	0
Bloemfontein, South Africa	102	26	5	3	99	62	40	19	83	76	18	8	97	0	0
CDC Zambia ADR	17	14	0	16	0	11	6	3	14	0	0	10	17	0	0
Durban, South Africa	58	51	12	0	58	25	33	0	51	7	29	22	46	12	0
KZN, South Africa	115	0	0	0	115	64	38	38	77	115	0	0	115	0	0
MSF Swaziland	26	26	25	0	26	16	10	6	20	0	13	13	0	17	8
OCTANE South Africa	16	16	16	16	0	16	0	16	0	0	7	0	0	0	14
PASER South Africa	11	10	11	0	0	0	0	0	0	0	0	0	0	0	0
PASER Zambia	23	23	21	23	0	10	0	0	18	0	0	14	0	9	0
**West/Central Africa**	**92**	**89**	**50**	**56**	**13**	**27**	**33**	**50**	**20**	**0**	**37**	**20**	**21**	**12**	**29**
ACTION Plus UP, Nigeria	21	20	0	0	0	0	0	0	0	0	0	0	0	0	0
ACTION, Nigeria	21	19	0	21	0	0	15	10	11	0	14	0	21	0	0
Lubumbashi, DRC	13	13	13	13	0	8	0	13		0	0	6	0	0	10
Doris Duke study,	13	13	13	0	13	7	6	4	9	0	5	8	0	7	0
Nigeria, Kanki	24	24	24	22	0	12	12	23	0	0	18	6	0	5	19

Supplementary Table 3: Information on drug resistance and baseline characteristics of participants by HIV-1 subtype. Note that subtype AG is also known as CRF_02

Subtype	N	TAM, N (%)	TDF resistance, N (%)	EFV or NVP resistance, N(%)	Lamivudine resistance, N(%)	NVP use, N(%)	FTC use, N(%)	Baseline CD4 (cells/mm3), median (IQR)	Baseline viral load (log10/ml), median (IQR)
A	90	21 (23.3%)	52 (57.8%)	73 (81.1%)	60 (66.7%)	65 (72.2%)	25 (27.8%)	113.0 (50.0 to 223.0)	5.6 (5.2 to 5.9)
AG/G	49	10 (20.4%)	38 (77.6%)	46 (93.9%)	44 (89.8%)	33 (67.3%)	37 (75.5%)	66.0 (30.0 to 126.0)	5.2 (4.7 to 5.5)
C	481	80 (16.6%)	293 (60.9%)	404 (84.0%)	305 (63.4%)	122 (25.4%)	87 (18.1%)	93.5 (34.0 to 159.0)	4.8 (3.6 to 5.5)
D	42	3 (7.1%)	30 (71.4%)	30 (71.4%)	31 (73.8%)	22 (52.4%)	23 (54.8%)	77.0 (25.5 to 178.5)	5.6 (5.0 to 5.8)
Other	50	1 (2.0%)	32 (64.0%)	42 (84.0%)	38 (76.0%)	28 (56.0%)	32 (64.0%)	92.0 (26.0 to 221.0)	5.4 (5.1 to 5.9)

Supplementary Figure 1: a) Scatter of study-level prevalence of NNRTI resistance and prevalence of TAM by region. Markers are weighted by study size. (Spearman's rho=0.62 p<0.0001); **b)** meta-analysis of odds ratios for NNRTI resistance in participants with TAM versus those without TAM within individual studies

a)

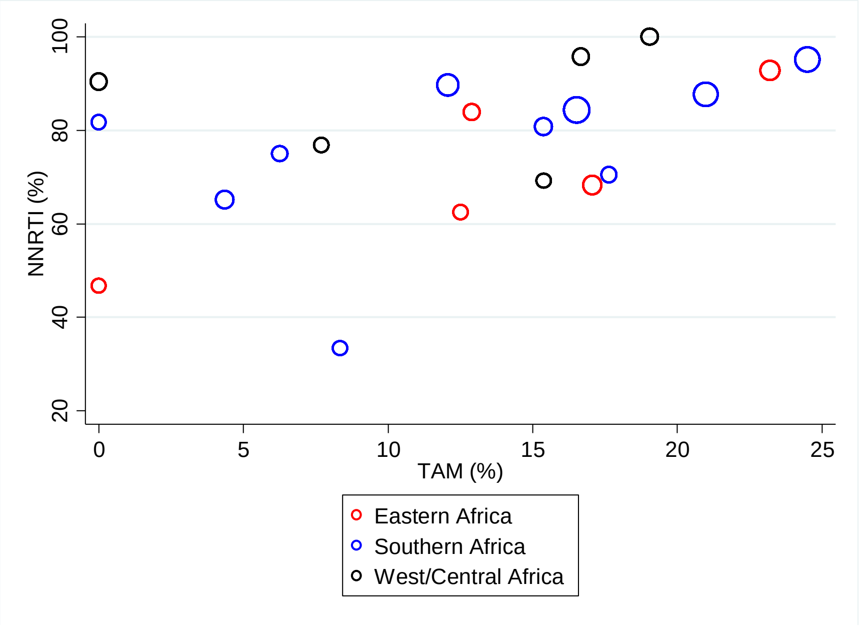

b)

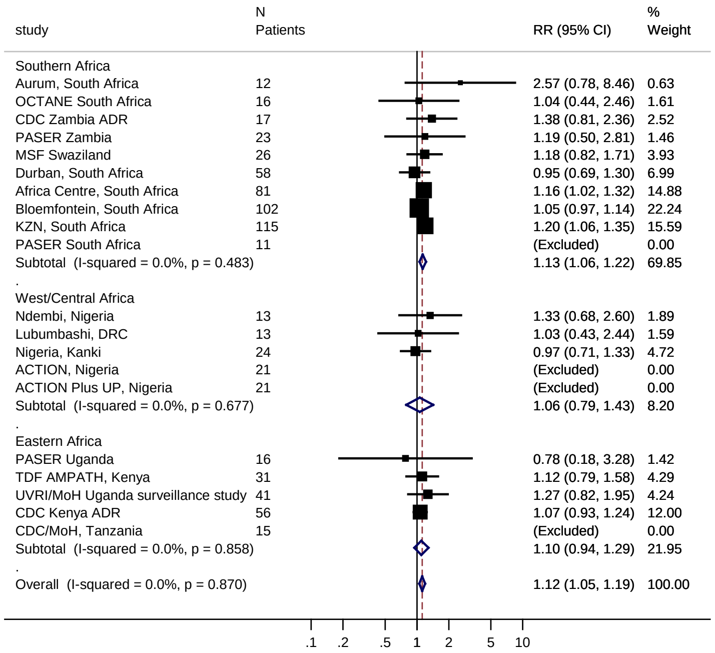

Supplementary Figure 2: a) Scatter of study-level prevalence of lamivudine resistance and prevalence of TAM by region. Markers are weighted by study size. (Spearman's rho=0.65 p<0.0001; **b)** meta-analysis of odds ratios for lamivudine resistance in participants with TAM versus those without TAM within individual studies

b) b)

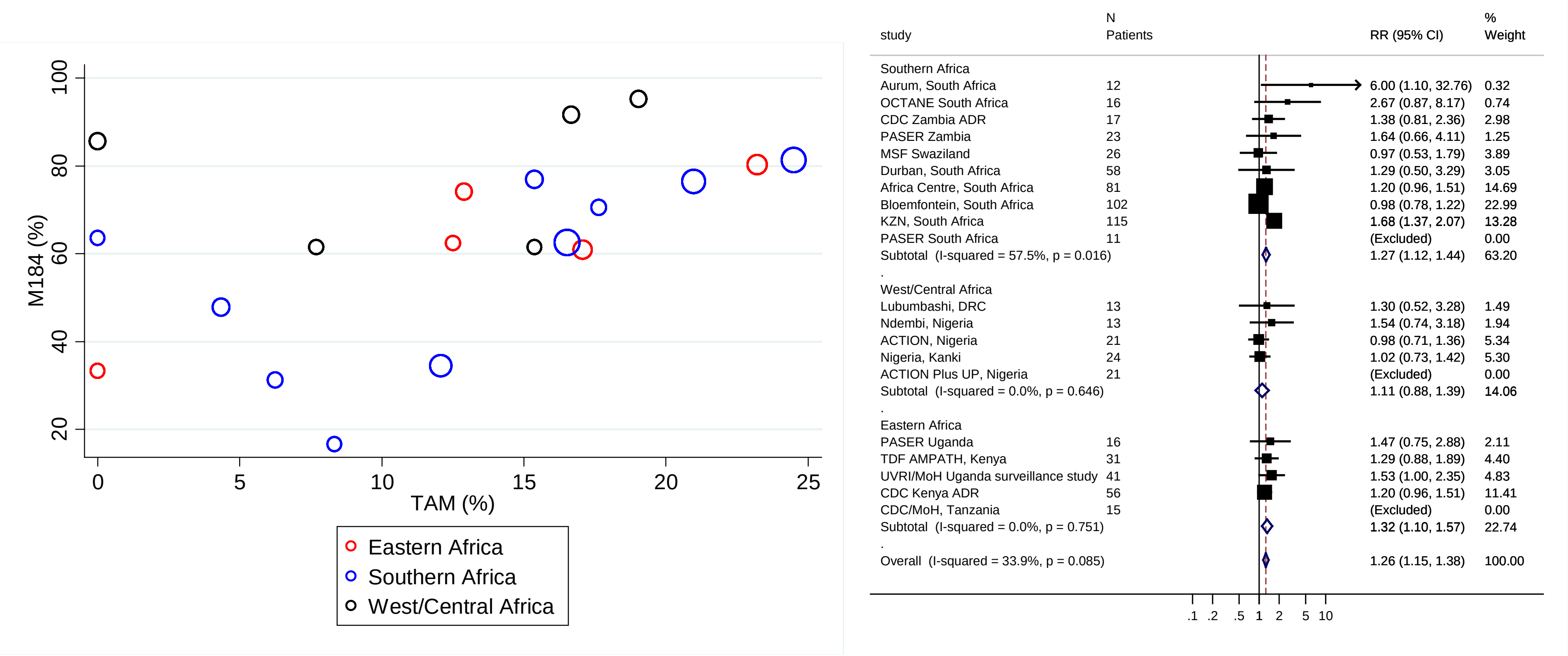

Supplementary Figure 3: Estimated prevalence of drug resistant mutations, sensitivity analyses including all participants from studies in sub-Saharan Africa (including those with <10 patients)

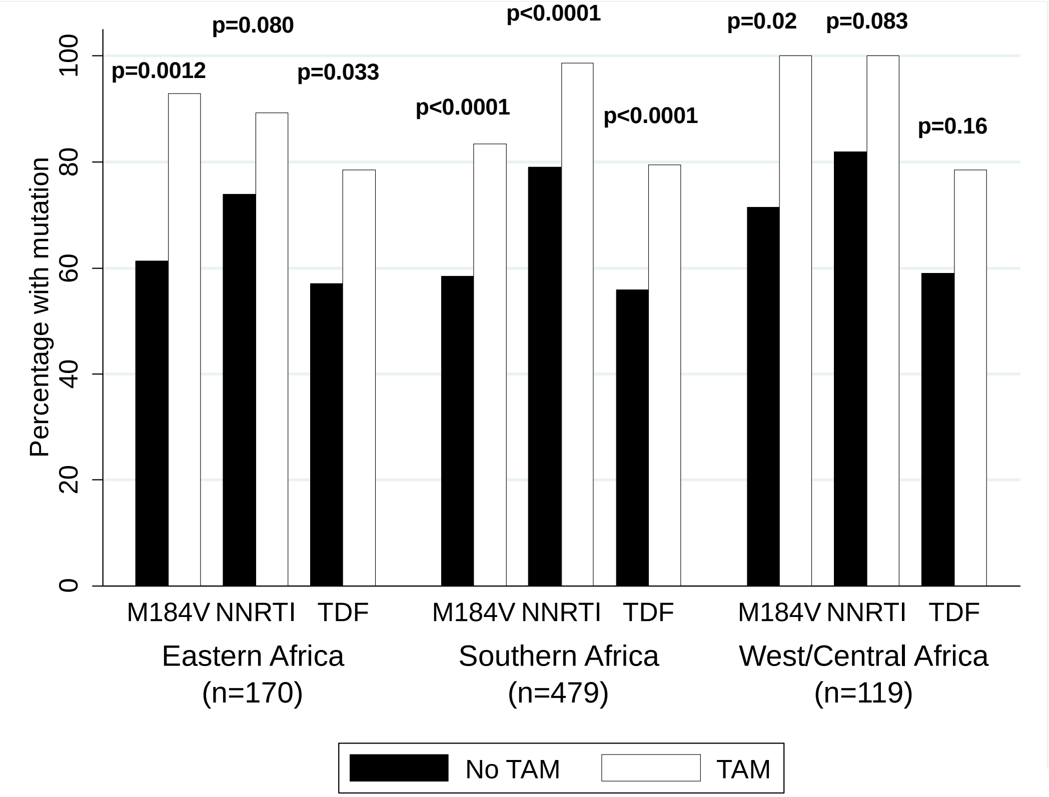

Supplementary Figure 4: Within study comparison of tenofovir resistance by presence or absence of TAMs; sensitivity analyses including all participants from studies in sub-Saharan Africa (including those with <10 patients)

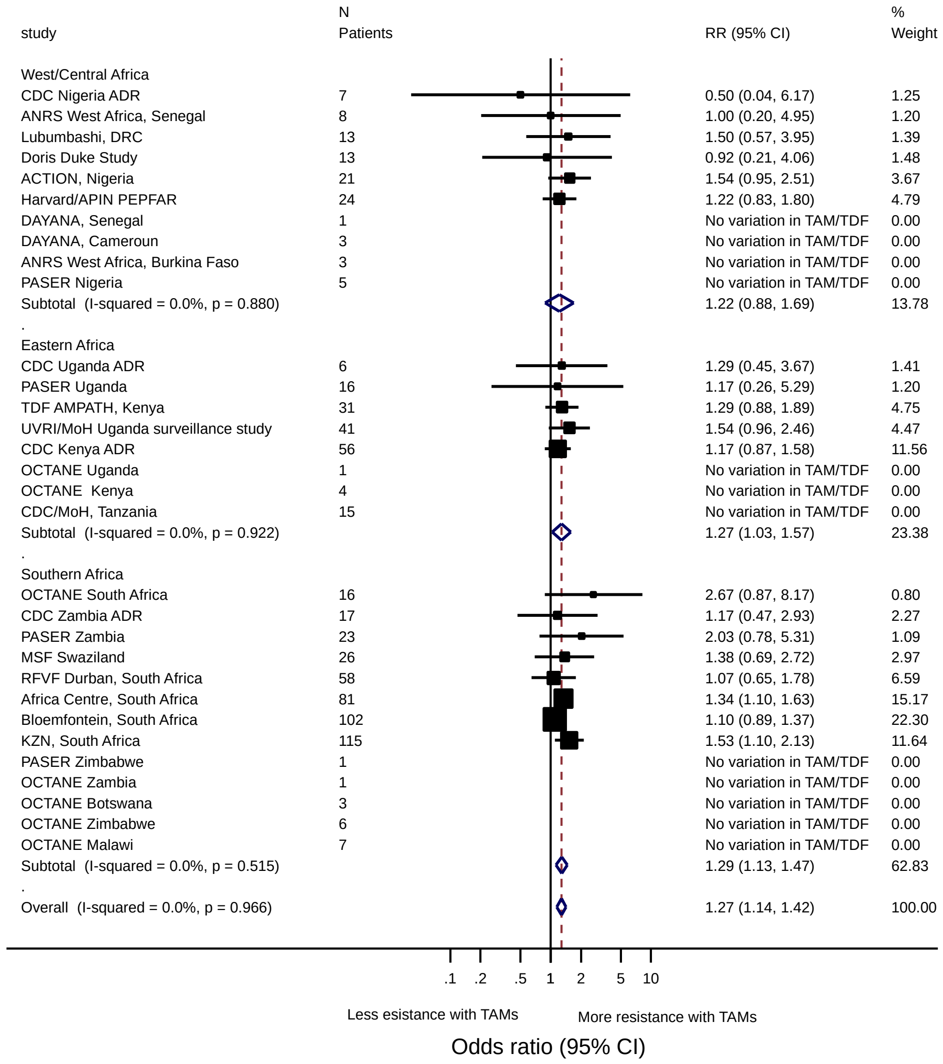

Study Groups

Uganda Virus Research Institute/Ministry of Health (UVRI/MoH) Uganda surveillance study: Fred Lyagoba, Tom Lutalo', Anne, Kapaata, Faith Nanyonga, Chris Parry, Norah Namuwenge, Robert Downing, The HIV Drug Resistance Working group and participants and study teams from the treatment centers at Masaka and Mbale regional referral hospitals and Nsambya Home-Care.

ACTG 5208 study team: Shahin Lockman, John Mellors, Michael Hughes, Fred Sawe, James McIntyre, Judy Currier. Research reported in this publication was supported by the National Institute of Allergy and Infectious Diseases of the National Institutes of Health under Award Number UM1 AI068634, UM1 AI068636 and UM1 AI106701. The content is solely the responsibility of the authors and does not necessarily represent the official views of the National Institutes of Health

RFVF: We would like to express our deepest admiration and appreciation for the patients who participated in the study and the work of the Sinikithemba Clinic at McCord Hospital in Durban, South Africa for their commitment to improve patient care and support research. The tremendous contributions on the part of the counselors, medical records staff, nurses, and medical officers have been essential to the success of this study. Sabelo Dladla, Michelle Gordon, Jane Hampton, Brent Johnson, Daniel Kuritzkes, Roma Maharaj, Darius McDaniel, Kristy Nixon, Claudia Ordonez, Melisha Pertab and Sifiso Shange provided vital assistance for the data collection and analysis.

Tanzanian, Kenyan and Ugandan Ministries of Health

The Harvard/AIDS Prevention Initiative in Nigeria (APIN) prevention, treatment and care program: Participating hospitals in this study included the University College Hospital, University of Ibadan, Ibadan, Lagos University Teaching Hospital, University of Lagos, Lagos, Jos University Teaching Hospital, University of Jos, and the Nigerian Institute of Medical Research, Lagos.

PEPFAR and CDC

Tanzanian, Nigeria and Kenyan Ministries of Health. Infectious Disease Institute, Uganda and Tropical Disease Research Centre, Zambia.

The Cross Sectional Survey of Acquired Drug Resistance Study at Sentinel Sites Study Team:

University of Maryland: Robert Redfield, Sekela Mwakyusa, Peter Memiah, Martine Etienne-Mesubi, Sandra Medina-Moreno, Andrew Kigombola, Sylvia Ojoo, Francesca Odhiambo,

Westat: Karen Megazzini, Annie Lo, Laura Alvarz-Rojas

Kenya: Sarah Masyuko, Shobha Vakil, Ibrahim Mohammed, David Kimanga, Maureen Kimani, and the Kenya National HIVDR working group

Tanzania/Zanzibar: Bonita Kilama, Mathias Abuya, Ben Rabiel, Sophia Mohamed, Jullu Boniphace, Geoffrey Somi, Ahmed Khatibu and the Tanzanian National HIVDR working group

CDC-Kenya: Lucy Nganga, Evelyn Ngugi, Andrea Kim, Jane Mwangi, Anthony Gichangi, Abraham Kitana, Frankline Onchiri, Frederick Miruka

CDC-Tanzania: Mary Kibona, Julius Muhumuza, Jennifer Ward, John Rogers, Duncan Donnay, Rama Mwiru, Godwin Munuo, Mohamed Mfaume, Eunice Mmari, Michelle Roland.

CDC-Atlanta: Tedd Ellerbrock, Laura Broyles, Jennifer Sabatier, Emilia Rivadeneira, Guoqing Zhang, Joshua R. DeVos, Nicolas Wager, Duping Zheng, Karidia Diallo and R. Suzanne Beard.

We would like to thank the following members of the TenoRes study team: Lou Halvas, Lameck Diero, Dominique Goedhals, Armand Bester, Soo-Yon Rhee, Michele Tang, Tobias F Rinke de Wit, Katherine Brooks, Henry Sunpath, Avelin Aghokeng, Simon Agolory and James M Juma.

ACTIONPlus Up: Charles Mensah, Patrick Dakum. Supported by The President's Emergency Plan for AIDS Relief through cooperative agreement (5U2GGH000925-03) from HHS/Centers for Disease Control and Prevention (CDC), Global AIDS Program.

AMPATH: We thank the study participants and AMPATH community, as well as Katherine Brooks, Allison DeLong, Maya Balamane, Marissa Reitsma, Emmanuel Kemboi, Millicent Orido, Mia Coetzer and Joseph Hogan for their help with patient enrollment, data generation and analyses in the original study.

The findings and conclusions in this article are those of the authors and do not necessarily represent the official position of the U.S. Centers for Disease Control and Prevention. Use of trade names is for identification purposes only and does not constitute endorsement by the U.S. Centers for Disease Control.

The Africa Centre drug resistance cohort was funded European Union (SANTE 2007 147–790), the US Centre for Diseases Control via CAPRISA (project title: Health Systems Strengthening and HIV Treatment Failure (HIV-TFC)) The data curation and research in this cohort was funded through a Medical Research Council flagship grant from the Republic of South Africa (MRC-RFA-UFSP-01-2013/UKZN HIVEPI) and by a Royal Society Newton Advanced Fellowship to T.d.O.
